# Diffuse Alveolar Hemorrhage as a Life-Threatening Feature of Juvenile Onset Systemic Lupus Erythematosus: A Case-Based Review

**DOI:** 10.31138/mjr.33.1.81

**Published:** 2022-03-31

**Authors:** Belde Kasap-Demir, Gülenay Bayram, Zuhal Önder-Siviş, Burçak Güneş-Tatlı, Berna Ayşe Anıl

**Affiliations:** 1 Department of Pediatrics Division of Nephrology & Rheumatology, İzmir Katip Çelebi University, İzmir, Turkey,; 2 Department of Pediatrics, Health Sciences University İzmir Tepecik Training and Research Hospital, İzmir, Turkey,; 3 Division of Hematology, Health Sciences University İzmir Tepecik Training and Research Hospital Department of Pediatrics, İzmir, Turkey,; 4 Department of Pediatrics Division of Pediatric Intensive Care, İzmir Katip Çelebi University, İzmir, Turkey

**Keywords:** diffuse alveolar hemorrhage, juvenile-onset systemic lupus erythematosus

## Abstract

Diffuse alveolar hemorrhage is an uncommon but serious complication of systemic lupus erythematosus (SLE). We reported a 17-year-old boy with idiopathic thrombocytopenic purpura, who admitted with pallor and petechiae. He had Coombs positive hemolytic anemia and thrombocytopenia (hemoglobin 6.2g/dL, platelets 10,000/mm^3^ and lactate dehydrogenase 1024U/L), cough, tachypnea, and desaturation in the room air. Chest radiograph revealed bilateral diffuse alveolar opacities and computed tomography showed bilateral diffuse alveolar infiltrates and ground-glass opacity consistent with pulmonary hemorrhage. Anti-nuclear antibody (ANA) was 1:640 with positive Ro and anti-phospholipid antibodies, low C3 and C4, but negative anti-double-stranded DNA. He was treated with pulse methylprednisolone followed by tapering doses of steroids and with 6 doses of intravenous cyclophosphamide once every two weeks followed by mycophenolate mofetil. He had no relapse in the following 3 years. The case was reported to emphasise this life-threatening complication of juvenile-onset SLE and we reviewed the literature.

## INTRODUCTION

Diffuse alveolar hemorrhage (DAH) is a life-threatening manifestation of systemic lupus erythematosus (SLE) first described in 1904 by Osler.^1^ It is characterised by sudden onset of dyspnea, shortness of breath, hemoptysis, and drop in serum hemoglobin levels accompanied by diffuse alveolar and interstitial infiltrates in radiographic examinations.^2^ It has been reported in 2–5% of cases of SLE, and the female-to-male ratio is approximately 6:1.^3,4^ It usually occurs in patients with an established diagnosis of SLE and the average time between diagnosis and DAH was reported as 1.8–7.1 years.^4^ However, it may be the presenting symptom in some cases or may even precede the diagnosis of SLE.^5^ The mortality rate may change between 23–92%.4 Diffuse alveolar hemorrhage has been usually reported in adult SLE patients. However, DAH in juvenile-onset SLE (jSLE) has been reported to be more mortal when compared to adults.^2^ It is very rare, and a distinct treatment of choice has not been established for DAH in jSLE. High dose steroids, cytotoxic agents, plasmapheresis, and intravenous immunoglobulin were reported to be effective. However, recurrences of this fatal complication may be seen despite aggressive treatment. In this study, we report our experience in a case with DAH and jSLE. In addition, we reviewed the jSLE cases with DAH reported in the literature and define treatment modalities and long-term effects.

## CASE REPORT

A 17-year old boy was admitted with fatigue and paleness to the pediatric hematology clinic, where he has been followed up with the diagnosis of refractory idiopathic thrombocytopenic purpura for the last 6 months. On his physical examination, he had pallor and widespread petechiae, more intensely on the lower extremities. His body weight and height were in the 50-75 p and his vital signs were normal except for tachycardia (120/min). There were inactive bleeding foci in the mouth and throat mucosa. The liver was approximately 2 cm palpable and spleen was non-palpable. There was no other pathological feature in physical examinations.

**Figure 1.**
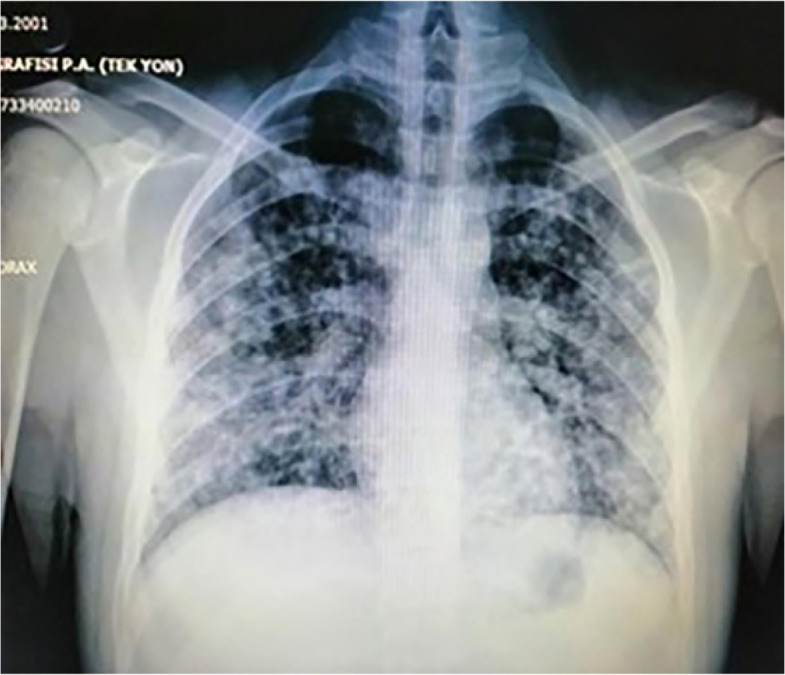
Bilateral diffuse alveolar opacities suggestive of pulmonary hemorrhage.

Initial laboratory investigations revealed hemoglobin 6.2 g/dL, white cell count 7400/mm3, platelets 10,000/mm3, C-reactive protein 14.2 mg/L, lactate dehydrogenase 1024 U/L, total bilirubin 1.59 mg/dL (N: 0.3-1.2) direct bilirubin 0.24 mg/dL (0–0.2). Renal function tests, electrolytes, and liver enzymes were in normal limits. Direct Coombs test and anti-immunoglobulin M was positive. He was hospitalised for Coombs positive hemolytic anemia and thrombocytopenia, was transfused with erythrocyte suspension, and IV fluid support was applied. On the second day of hospitalisation, he began to cough. He had tachypnea (35/min) and he was desaturated in the room air. He had bilateral diffuse rhonchi and rales in the lung auscultation. High-flow nasal cannula oxygen support was instituted in the intensive care unit and vancomycin, ceftriaxone, clarithromycin, and oseltamivir were added to the treatment. Chest radiograph revealed bilateral diffuse alveolar opacities (**Figure 1**) and thorax computed tomography showed bilateral diffuse alveolar infiltrates and extensive ground-glass opacity consistent with pulmonary hemorrhage. In advanced investigations, the antinuclear antibody (ANA) was 1:640 with positive Ro antibodies and anti-double-stranded DNA was negative. Both C3 and C4 were low; C3 was 59.3 mg/dL (N: 79–152) and C4 was 8.54 mg/dL (16–38). Anti-cardiolipin IgG and IgM were >120 U/mL (negative <12); anti beta-2 glycoprotein 1 IgA, IgM and IgG were 98.61, >200 and >200 RU/mL (negative<20) respectively. Lupus anticoagulant could not be determined, however, activated partial thromboplastin time (aPTT) was 19 sec (N:21–36). Ebstein-Barr virus, cytomegalovirus, parvovirus, TORCHES group, hepatitis B, C and HIV serology were all negative. Urinalysis was normal.

After bone marrow aspiration, treatment was started with pulse methylprednisolone (PMP) at a dose of 30 mg/kg/day in addition to 1 g/m^2^ IV cyclophosphamide (CYC). A significant decrease in tachypnea and respiratory distress, an increase in oxygen saturation was observed on the first day. A total of 5 doses of PMP was planned for both alveolar hemorrhage and hemolytic anemia and thrombocytopenia. A significant radiological improvement in chest X-ray was observed on the 5th day of treatment. Hemoglobin increased to 9.7 g/dL, however, platelets were still 14,000/mm^3^. Oral steroid treatment was instituted and gradually decreased in the follow-up. Cyclophosphamide treatment was planned as in Euro Lupus protocol (500 mg every fortnight). Platelets dramatically began to rise after the second dose of CYC. After the 6th dose of CYC, 750 mg mycophenolate mofetil twice a day was initiated along with 5 mg daily prednisolone and hydroxychloroquine. The patient did not relapse during the following 2-year period and he is in both clinical and laboratory remission at the end of the second year.

### Search Strategy and Results

We searched MEDLINE/PubMed, Google, Web of Science databases for original articles, reviews, case reports, and letters published in English. We used the keywords “systemic lupus erythematosus” and “juvenile” or “children” and “diffuse alveolar hemorrhage” or “pulmonary hemorrhage”. Titles, abstracts, and full texts were screened and the data of patients ≤18years have been collected from case series including both children and adults. The final search was established by the first author.

In our literature review, there were 45 children with DAH. The demographic and clinical findings at admission were summarised in **Table 1**. The laboratory findings were summarised in **Table 2** and treatment modalities for children with jSLE and DAH were summarised in **Table 3**.

**Table 1.** Demographic and clinical findings of the patients with DAH.

**Authors (Year) (Reference)**	**Cases**	**Age at onset of SLE**	**Age at onset of AH**	**Duration of SLE**	**Gender (F)**
Eagen (1978)^10^	1	16	16 y	4.5m	F
	2	18	16y	16m prior	M
Ramirez (1984)^5^	1	6	7	1y	F
	2	9	9	1m	F
	3	13	13	0	M
	4	14	14	1m	F
Fukuda (1994)^14^	1	12	12	2m	F
Uziel Y (1997)^29^	1	13	13	0	F
Liu (1998)^25^	1	15	15	2m	F
	2	8	10	2y	F
	3	17	18	1m	F
Chang (2002)^15^	1	14	15	16m	F
Çiftçi E (2004)^3^	1	12	18	6y	F
	2	13	22	9y	F
Beresford (2005)^11^	1	14	14	0	M
Canas (2007)^8^	1	2	4	2y	F
	2	6	6	0	M
Stamp L (2008)^30^	1	13	13	0	F
Moradinejad (2009)^19^	1	ND	14	ND	M
	2	ND	15	ND	F
Vijatov-Djurig (2010)^20^	1	17	17	0	F
Araujo (2012)^2^	13	12.7±4.2	15.3±2.7		F: 10/13 (77%)
Kimura (2015)^21^	1	14	14	1m	F
Bhadauria (2015)^7^	1	10	10	0	F
Singia (2016)^16^	7		14^3 – 15^		m (5; 71%)
Cucuzza (2017)^4^	1	14	14	0	M
Balcı (2019)^18^	1	ND	ND	ND	F

**Table 2.** Laboratory findings of the patients.

**Authors**	**Cases**	**ANA**	**Anti-dsDNA**	**Anti-cardiolipin IgG/M**	**C3**	**C4**	**Hemolytic anemia**	**Leukopenia**	**Thrombocytopenia**	**Renal involvement**
Eagen (1978)^10^	1	1/500	1/640	ND	low	low	+	ND	-	+
	2	1/500	1/16	ND	low	ND	+	+	+	+
Ramirez (1984)^5^	1	1/640	ND	ND	low	low	ND	ND	ND	ND
	2	1/80	ND	ND	ND	ND	ND	ND	ND	ND
	3	ND	ND	ND	low	low	ND	ND	ND	ND
	4	+	ND	ND	ND	ND	ND	ND	ND	+
Fukuda (1994)^14^	1	1/320	+	ND	low	ND	-	-	+	+
Uziel Y (1997)^29^	1	+ (intially)	+	ND	ND	ND	ND	ND	ND	-
Liu (1998)^25^	1	1/320	ND	-	low	ND	ND	ND	ND	+
	2	-	ND	+	low	ND	ND	ND	ND	+
	3	1/640	ND	ND	low	ND	ND	ND	ND	+
Chang (2002)^15^	1	ND	-	-	low	Low	-	-	+	+
Çiftçi E (2004)^3^	1	+ (intially)	+ (intially)	ND	low (initially)	low (initially)	-	ND	ND	+
	2	+ (intially)	+ (intially)	ND	low (initially)	low (initially)	-	ND	ND	+
Beresford (2005)^11^	1	+	-	-	ND	ND	+	ND	+	+
Canas (2007)^6^	1	1/320	+	ND	low	low	-	-	-	+
	2	1/320	ND	ND	low	low	ND*	ND*	ND*	-
Stamp L (2008)^30^	1	1/1280	-	+ (weak)	low	low	+	-	-	-
Moradinejad (2009)^19^	1	1/1280	+	ND	low	low	-	-	-	+
	2	1/160	+	ND	low	low	-	-	-	+
Vijatov-Djurig (2010)^20^	1	+	+	ND	low	low	-	+	-	+
Araujo (2012)^2^	13	ND	+(85%)	1(8)	ND	ND	4 (31%)	7 (54)	3 (23)	10/13 (77%)
Kimura (2015)^21^	1	1/360	ND	ND	ND	ND	-	+	ND	+
Bhadauria (2015)^9^	1	1/80	>200	+ (strong)	low	low	+	+	+	+
Singia (2016)^16^	7	ND (?)	+ n:6, 86%	ND	ND	ND	anemia (100%)		(5; 71%)	n:6, 86%
Cucuzza (2017)^4^	1	1/1280	+	+	low	low	anemia (+)hemolytic?	-	-	-
Balcı (2019)^18^	1	ND	ND	ND	ND	ND	ND	ND	ND	+

ND: not determined,

*: reported as cytopenia

**Table 3.** Treatment modalities and prognosis of patients with DAH.

**Authors**	**Cases**	**PulseMP**	**Oral pred**	**CYC**	**IVIg**	**Plasmapheresis**	**AZA**	**MMF**	**Survival**	**Recurrence of DAH**
Eagen (1978)^10^	1	+	+	+	-	-	-	-	+	-
	2	-	+	+	-	-	-	-	-	-
Ramirez (1984)^5^	1	ND	ND	ND	ND	ND	ND	ND	+	+
	2	+	+	ND	ND	ND	ND	ND	+	ND
	3	ND	ND	ND	ND	ND	ND	ND	-	ND
	4	ND	ND	ND	ND	ND	ND	ND	-	ND
Fukuda (1994)^13^				+ (1g/month)						
	1	+	+	(7 months)	-	+	-	-	+	-
Uziel Y (1997)^29^	1	+	+	+	-	-	-	-	+	-
Liu (1998)^25^	1	+	+	+	-	-	-	-	-	-
	2	+	+	+	-	-	-	-	-	+ (5 times)
	3	-	+	-	-	-	-	-	+	-
Chang (2002)^15^	1	+	+	-	-	+	-	-	-	Died
Çiftçi E (2004)^3^	1	+	-	-	-	-	-	-	+	ND
	2	+	-	-	-	-	-	-	+	ND
Beresford (2005)^11^	1	+	+	+ (6 months)	-	-	-	-	+	-
Canas (2007)^8^	1	+	+	+ (750mg/m^2^)	-	-	-	-	+	-
	2	+	+	+ (750mg/m^2^)	-	+	-	-	-	-
Stamp L (2008)^30^	1	-	+	-	-	-	+ *	+	+	+ (initially)
Moradinejad (2009)^19^	1	+	+	+ (1g monthly )	+	+	-	+ *	+	+ (10 days after the first attack)
	2	+ *	+	+ *	+ *	+ *	-	+ *	+ (10 days after MMF)	+ (15 days after the first attack)
Vijatov-Djurig (2010)^20^	1	+	+	+ (1g/month for 6m)	-	-	-	-	+	-
Araujo (2012)^2^									+ 4	ND
	13	13 (100%)	13 (100%)	9 (69%) frequency?	5 (39%)	2 (15)	-	-	(31%)	
Kimura (2015)^21^	1	+	+	+ (1 single dose)	+ (2g/kg)	+	-	-	+	-
Bhadauria (2015)^9^	1	-	+ (later)	-	+	+	-	+ (later)	+	ND
Singia (2016)^16^		6 (86%)	7 (100%)	5 (71%)	2 (29%)	2 (29%)	1 (14%)	-	+ 6	
	7								(86%)	-
Cucuzza (2017)^4^	1	+	+	+ *	-	-	-	+ (maintenance)	+ (even after second DAH)	+
Balcı (2019)^18^	1	+	+	+	-	-	-	-	-	-

*: at the time of the second attack,

**: two courses of EUROLUPUS protocol.

## DISCUSSION

Diffuse alveolar hemorrhage is an unpredictable and devastating disorder characterised by intraalveolar bleeding from damaged pulmonary vasculature with a mortality rate of 20%.^6^ Differential diagnosis of non-infectious causes of DAH includes coagulopathy; exposure to several drugs including abciximab, amiodarone, anticoagulants, carbimazole, crack cocaine, leflunomide, nitrofurantoin, penicillamine, propylthiouracil, sirolimus, TNF-α antagonist, and trimellitic anhydride; mitral stenosis or regurgitation; and autoimmune diseases.^6,7^ The autoimmune diseases associated with DAH include granulomatosis with polyangiitis, microscopic polyangiitis, Goodpasture syndrome, antiphospholipid syndrome, isolated pauci-immune pulmonary capillaritis, idiopathic pulmonary hemosiderosis, and SLE. Although DAH mostly occurs in young adults with autoimmune diseases, its association with those diseases other than SLE has rarely been reported in children.^6,7^ In this paper, we briefly reviewed the literature relevant to pulmonary hemorrhages associated with jSLE.

In their study, Araujo et al. defined DAH with regard to at least three of the following: pulmonary symptoms (hemoptysis, dyspnea, hypoxemia, tachycardia, and/or cough), new infiltrates on chest radiograph or computed tomography; a sudden drop of hemoglobin of at least 1.5 g/dL without other sources of bleeding; bloody return and hemosiderin-laden macrophages in bronchoalveolar lavage^2^ and they excluded cases with coagulopathies, pulmonary edema, pulmonary bleeding due to other known causes, pulmonary embolism and bronchiectasis.^2,8^ However, Cucuzza et al. defined DAH only by the presence of three major components including clinical signs, a drop of hemoglobin, and diffuse infiltrates on chest imaging.^4^ Our case had both pulmonary symptoms and radiological evidence for DAH and a sudden drop of hemoglobin was associated also with hemolytic anemia, however, bronchoalveolar lavage was not performed. When we review the cases with jSLE associated DAH in the literature, we have found nine more patients having hemolytic anemia along with DAH.^2,9–11^

Some patients may have DAH at the first bout of active SLE or even present with DAH, while others display findings in the follow-up. Eight of 45 cases (17%) presented with DAH and 6 cases (13%) had DAH in the first 2 months of SLE diagnosis. Our case had immune thrombocytopenia for the last 6 months; however, he was diagnosed upon DAH concurrent with hemolytic anemia. In terms of differential diagnosis, the patient denied any exposure to the abovementioned drugs and tests for coagulation, cardiac abnormalities, and other autoimmune diseases were negative.

Thrombocytopenia may cause life-threatening hemorrhage as a direct result of low platelets. Besides, thrombocytopenia was found to be associated with hemolytic anemia, anti-phospholipid syndrome (APS), neuropsychiatric disease, and renal involvement. Thrombocytopenia in the setting of SLE, at least in some patients, is associated with anti-phospholipid (aPL) antibodies.^12^ Our patient with thrombocytopenia also had high titers of aPL antibodies but had no other findings of APS. Usually, 30% to 40% of SLE patients have aPL antibodies, however, only 10% progress to APS, and 1% of those with APS lead to catastrophic APS.^13^ The frequency of DAH among patients with APS has been reported as <1–21%.^9^ In the literature, almost 13 more cases were reported to have thrombocytopenia,^2,9–11,13–16^ however, only a few have additional aPL antibodies and aPL syndrome.^2,9^ Anti-nuclear antibody and anti-DNA tests were positive and complement levels were low in most of the cases. Anti-Sm, anti-Ro, and anti-La tests were rarely reported and found positive in the literature (**Table 2**).

Although active nephritis is a major risk factor for DAH in SLE patients, our patient had no renal disease either at presentation or in the follow-up. Of the 76% patients we found in the literature had renal involvement along with DAH^2,3,5,8–11,14–21^ (**Table 2**). A high index of suspicion is necessary for a correct diagnosis for DAH and prompt and aggressive treatment approach is of paramount importance. An optimal management approach has not been established for DAH yet. Mechanical ventilation is needed in most of the cases. Pulse methylprednisolone is suggested to be used as soon as possible.^17^ Following oral corticosteroids, CYC pulses followed by mycophenolate mofetil (MMF) may be the treatment of choice. Plasmapheresis, IVIg, or rituximab may also be effective in resistant cases.^22^ Pulmonary administration of human-recombinant activated factor VIIa may be effective for active pulmonary bleeding as well.^22,23^

Even only PMP-provided survival in some patients whereas more aggressive therapy might result in death in others. A combination of PMP and CYC as initial treatment was used for DAH in nearly 10 cases with jSLE, as we used in our patient. CYC was used just as a single dose or a total of 6 doses and the dose may be changed from 500 to 1000mg per m^2^ for each dose with an interval of a fortnight or a month. In 3 cases reported much earlier, CYC has been administrated orally instead of intravenous pulse doses and two of them did not survive. High doses of corticosteroids and pulse cyclophosphamide therapy resulted in rapid improvement of respiratory function in our patient. We preferred to continue with MMF after CYC. Treatment modalities for children with jSLE and DAH were summarised in **Table 3**.

Diffuse alveolar hemorrhage has a mortality rate ranging from 36% to 90% (17,24). 16 of the 45 cases (36%) with jSLE associated DAH were expired (**Table 3**). The considered underlying pathogenic mechanism was microangiitis, particularly alveolar capillaritis, and immune complex deposition in pulmonary parenchyma.^25–27^ The literature is not only limited to these cases. Nadorra et al. reported a study including pulmonary autopsy findings of 26 patients diagnosed with jSLE.^28^ The perspective of the study was different from the other series. They reported 18 cases with alveolar hemorrhage in histological examination that was mild or focal in 13 and moderate to massive in 5. In 9 of 26 cases, the cause of death was alveolar haemorrhage.^28^ We could not include those cases since the clinical findings of cases with alveolar haemorrhage were not separately evaluated.

## CONCLUSION

In conclusion, DAH is a rare and potentially lethal complication of SLE that sometimes may occur at an early stage of the disease. A high index of suspicion is necessary for the diagnosis and rapid treatment may be life-saving in DAH. Pulse methylprednisolone and CYC may be sufficient as seen in our patient, however, more aggressive treatment would be necessary in more severe cases. We reported this case to draw attention to this life-threatening complication of jSLE that causes acute respiratory distress and to review the literature.

## ABBREVIATIONS

ANA: Antinuclear antibody
aPL: Anti-phospholipid
APS: Anti-phospholipid syndrome
aPTT: Activated partial thromboplastin time
CYC: Cyclophosphamide
DAH: Diffuse alveolar hemorrhage
jSLE: Juvenile-onset systemic lupus erythematosus
MMF: Mycophenolate mofetil
PMP: Pulse methylprednisolone
